# Duodenum-Preserving Pancreatic Head Resection for Benign and Premalignant Tumors—a Systematic Review and Meta-analysis of Surgery-Associated Morbidity

**DOI:** 10.1007/s11605-023-05789-4

**Published:** 2023-09-05

**Authors:** Hans G. Beger, Benjamin Mayer, Bertram Poch

**Affiliations:** 1 c/o University of Ulm, Albert-Einstein-Allee 23, 89081 Ulm, Germany; 2 Institute for Epidemiology and Medical Biometry, Ulm University, Ulm, Germany; 3 Centre for Oncologic, Endocrine and Minimal Invasive Surgery, Donau-Klinikum Neu-Ulm, Neu-Ulm, Germany

**Keywords:** Benign pancreatic head tumors, Cystic and neuroendocrine neoplasms of the pancreas, Duodenum-preserving pancreatic head resection, Pancreatoduodenectomy

## Abstract

**Background:**

Pancreatic benign, cystic, and neuroendocrine neoplasms are increasingly detected and recommended for surgical treatment. In multiorgan resection pancreatoduodenectomy or parenchyma-sparing, local extirpation is a challenge for decision-making regarding surgery-related early and late postoperative morbidity.

**Methods:**

PubMed, Embase, and Cochrane Libraries were searched for studies reporting early surgery-related complications following pancreatoduodenectomy (PD) and duodenum-preserving total (DPPHRt) or partial (DPPHRp) pancreatic head resection for benign tumors. Thirty-four cohort studies comprising data from 1099 patients were analyzed. In total, 654 patients underwent DPPHR and 445 patients PD for benign tumors. This review and meta-analysis does not need ethical approval.

**Results:**

Comparing DPPHRt and PD, the need for blood transfusion (OR 0.20, 95% CI 0.10–0.41, *p*<0.01), re-intervention for serious surgery-related complications (OR 0.48, 95% CI 0.31–0.73, *p*<0.001), and re-operation for severe complications (OR 0.50, 95% CI 0.26–0.95, *p*=0.04) were significantly less frequent following DPPHRt. Pancreatic fistula B+C (19.0 to 15.3%, *p*=0.99) and biliary fistula (6.3 to 4.3%; *p*=0.33) were in the same range following PD and DPPHRt. In-hospital mortality after DPPHRt was one of 350 patients (0.28%) and after PD eight of 445 patients (1.79%) (OR 0.32, 95% CI 0.10–1.09, *p*=0.07). Following DPPHRp, there was no mortality among the 192 patients.

**Conclusion:**

DPPHR for benign pancreatic tumors is associated with significantly fewer surgery-related, serious, and severe postoperative complications and lower in-hospital mortality compared to PD. Tailored use of DPPHRt or DPPHRp contributes to a reduction of surgery-related complications. DPPHR has the potential to replace PD for benign tumors and premalignant cystic and neuroendocrine neoplasms of the pancreatic head.

**Supplementary Information:**

The online version contains supplementary material available at 10.1007/s11605-023-05789-4.

## Introduction

Pancreatoduodenectomy (PD) is the worldwide surgical standard for pancreatic head and periampullary cancer treatment. Due to a high level of standardization, a high quality of ICU management, the use of non-operative interventions for complications, and surgical expertise in many centers, Whipple resection and pylorus-preserving pancreatoduodenectomy (PPPD) are considered with increasing acceptance as the appropriate surgical treatments for benign tumors and premalignant, cystic, and neuro-endocrine neoplasms of the pancreatic head.^1, 2^ However, multi-organ resection poses substantial risks for surgery-related complications, hospital mortality, and long-term metabolic morbidity. Recently published results of large international mono- and multi-institutional studies of PD for benign tumors displayed an in-hospital mortality of 2–4% and a 90-day mortality above 4%.^1–8^ Pancreatic endocrine and exocrine dysfunctions have been assessed in the long-term outcome after PD for benign tumor. Data with high clinical evidence revealed that postoperative new-onset diabetes mellitus (DM) was observed in 14–20% of patients and new-onset pancreatic exocrine insufficiency (PEI) in 34–45% of patients.^9–13^ Resection of the duodenum and first jejunal loop is the main cause of the long-term endocrine and exocrine metabolic morbidity after PD.^14^

Symptomatic or clinically silent benign tumors and cystic and neuro-endocrine neoplasms (PNETs) of the pancreas are increasingly detected due to the expanded use of advanced cross-sectional imaging tools for the diagnosis of abdominal complaints. In high-volume centers, the prevalence of pancreatic cystic and neoplastic lesions has reached an average of 8% of an adult population in Western countries.^15^ The most common pathology encompasses the diagnosis of intraductal papillary mucinous neoplasm (IPMN), mucinous cystic neoplasm (MCN), solid pseudopapillary neoplasm (SPN), and serous cystic adenoma (SCN). IPMN and SPN are located predominantly in the pancreatic head and are prevalently found in males and young females, respectively. Neuroendocrine neoplasms are detected in approximately 2% of all pancreatic tumors.^16^ The diagnostic rate of PNETs comprising non-functional and functional neoplasm continues to rise, likely secondary to the frequent use of high-resolution imaging diagnostics. In centers with high caseloads of pancreatic surgery, approximately 15–20% of all pancreatic resections are performed for benign tumors or premalignant cystic neoplasm or PNETs.^17^

The development and increasing use of parenchyma-sparing, local resection of pancreatic tumors—tumor enucleation (TE),^18^ duodenum-preserving pancreatic head resection (DPPHR),^19, 20^ and pancreatic middle segment resection (PMSR)^21^—parallels the increase in the number of patients with symptomatic or asymptomatic benign neoplasms requiring surgical treatment.

DPPHR has the advantage of conservation of the duodenum and reduced loss of pancreatic and biliary tissues. Accordingly, new-onset DM and new-onset PEI were assessed to be below of 6%, while in most patients endocrine and exocrine functions were measured after DPPHR to be at the preoperative level.^11^

While a low rate of metabolic dysfunctions following total DPPHR (DPPHRt) has been reported with high clinical evidence by many institutions,^11, 22–27^ data of clinical evidence for procedure-related early postoperative morbidity following DPPHR is lacking. Consequently, this systematic review and meta-analysis aim to evaluate the pattern of early postoperative surgery-related morbidity and the level of evidence when comparing DPPHR and PD. The hypothesis was that DPPHR applied for benign tumors ensures the cure of patients associated with a low risk for procedure-related surgical morbidity. The primary endpoints were the metrics for the severity of the surgical procedures and early postoperative outcome criteria, defined as severe or serious surgery-related complications corresponding to Clavien-Dindo grade ≥ III, and in-hospital mortality.

## Material and Methods

### Search Strategy

We conducted a comprehensive literature search of the PubMed/Medline, Embase, and Cochrane databases. For PubMed, a search for Medical Subject Headings (MeSH-Terms) was used; for Embase and Cochrane, searches with Emtree terms and MeSH-Terms were performed, respectively, including a text word search for surgical techniques. Additionally, a text word search for pancreatic resection techniques including duodenum-sparing head resection and pancreatoduodenectomy for benign tumors was performed. The following search terms were used: duodenum-preserving pancreatic head resection, parenchyma-sparing surgery for pancreatic head tumors, pancreatoduodenectomy for benign tumors, Whipple resection for cystic neoplasm, pancreatic head resection with segment resection of the duodenum, local resection of periampullary tumors. Studies reporting limited surgery for cystic neoplasms, neuro-endocrine tumors of the pancreatic head, or low-risk periampullary tumors were included in the selection process. The preoperative and final histological diagnosis of benign tumors of the pancreatic head included IPMN, MCN, SPN, and SCA; non-functional and functional PNETs; periampullary tumors; inflammatory tumors of chronic pancreatitis; and “other” tumors.

**Fig. 1:**
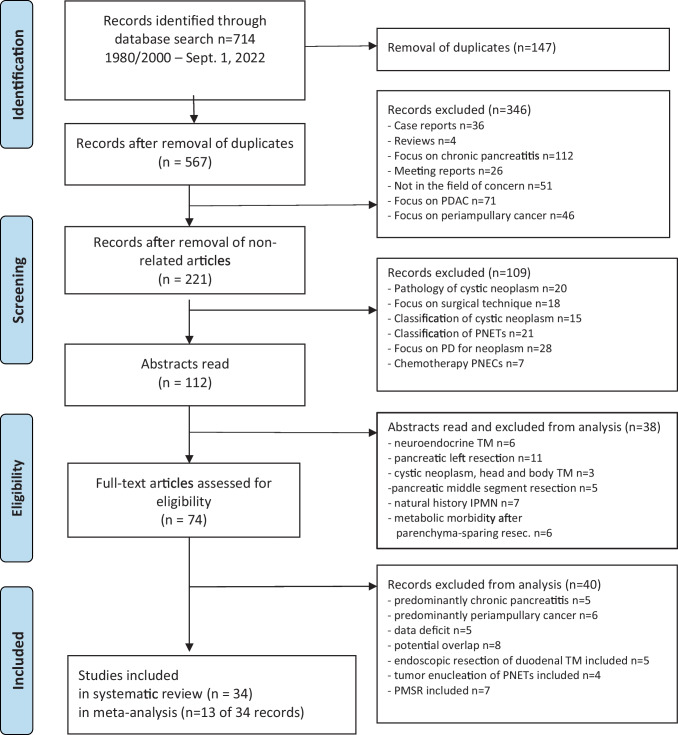
PRISMA flow diagram on the selection process of studies

The search results for identifying relevant publications are presented in Fig. 1. The following studies were excluded: case reports, case series up to four patients, reports of assessments of metabolic functions after pancreatic head surgery, and studies including advanced pancreatic head cancer. Figure 1 shows the PRISMA flow diagram of the selection process.^28^ The publications were checked for cross-references to identify eligible additional reports that were not identified by the primary search items. Differences were resolved by mutual agreement between two authors (HB, BP).

### Evaluation of Methodological Quality of Studies

The methodological quality of the 34 studies finally included in the systematic review and meta-analysis was assessed using the Critical Appraisal Skills Programme of the Oxford Centre for Evidence-Based Medicine.^29^ The manuscripts were evaluated according to this program for the level of evidence; specifically, criteria for selection and measure bias, and applicability, were assessed for each study. Additionally, the Newcastle-Ottawa Scale (NOS) was used to assess the quality of the controlled, prospective, and retrospective cohort studies, ensuring an objective evaluation of the most basic quality aspects of non-randomized cohort studies with regard to selection criteria, case definition, representativeness of cases, selection of controls, comparability of study groups, and assessment of outcome variables.^30^ Cohort studies with scores of 8 or 9 were considered having good-to-high levels of evidence and were included in the analysis (Tables 1 and 2).

**Table 1:** Baseline data and quality assessment of the review group. Duodenum-preserving pancreatic head resection (DPPHR) for benign tumors, premalignant cystic and neuroendocrine neoplasms and low-malignant periampullary tumors of the pancreatic head

Author/reference no.	Publ. year	Study period	Total pats *N*	Age Years	M/F	DPPHR total/partial	Type of cohort study	Quality assessment	
				Mean	*n*/*n*			Oxford evidence	NOS
Cai^31^	2021	2019	24	43	8/15	Total^†^	Retrospective	2c	8
Hong^32^	2020	2016–2019	22	46.7±16.2	6/16	Total 17^†^ Partial 5^†^	Prospective	2c	9
Snajdauf^33^	2019	1994–2015	13	14.9	8/13	Total	Prospective	3a	8
Cao^34^	2019	2016–2017	12	37.3	2/10	Total^†^	Prospective	3b	8
Milanetto^35^	2016	1991–2015	8	33	5/3	Partial	Prospective	2b	7
Yuan^36^	2016	2006–2013	12	42.3	5/7	Partial	Prospective	3b	8
Thomas^37^	2015	2008–2013	5	64	ND	Partial^†^	Prospective controlled	2c	8
Kozlov^38^	2014	ND*	16	ND	ND	Partial	Retrospective controlled	3a	7
Tsuchikawa^39^	2013	1994–2011	21	61	8/13	Total	Prospective	2c	8
Suzuki^40^	2013	2000–2012	5	54.5	1/4	Total	Retrospective	3a	8
Nakaghori^41^	2010	1994–2007	15	64	13/2	Partial**	Retrospective	3a	7
Beger^42^	2008	1982–2006	15	44	6/9	Total 11 Partial 4	Prospective	2c	8
Xiong^43^	2007	2001–2006	22	49.7	9/13	Partial	Prospective	2c	8
Fernandez-Cruz^44^	2006	1995–2006	8	65	ND	Total 4 Partial 4	Prospective controlled	2c	8
Murakami^45^	2004	ND	8	63±13	7/1	Total	Retrospective	3b	7
Hirano^46^	2004	1989–1998	13	59	9/4	Total	Retrospective	3c	7
Takada^47^	2004	1988–2002	26	53.3/59.1	15/11	Total	Prospective	2c	7
Yamaguchi^48^	2001	ND	6	54.5±4.6	4/2	Partial	Prospective controlled	2c	8
Isaji^49^	2001	1996–1999	18	ND	ND	Total	Retrospective	3a	7
Imaizumi^50^	1995	1989–1993	20	52.5	10/10	Total	Prospective controlled	2a	9
Harada^51^	1994	1989–1991	15	ND	ND	Total	Prospective controlled	2c	8

*ND no data

**Partial head resection processus uncinatus

^†^Laparoscopic DPPHR

**Table 2:** Baseline data and quality assessment of studies included in meta-analysis. Comparison of pancreatoduodenectomy (PD) and duodenum-preserving pancreatic head resection (DPPHR) for benign tumors, premalignant cystic and neuroendocrine neoplasms and low-malignant periampullary tumors of the pancreatic head

Author	Publication year	Study period	Total patients	Age	Gender	PD		DPPHR		Type of cohort study	Quality assessment	
						Patients	Type	Patients	Type		Oxford Evidence	NOS
			*N*	Years mean	M/F *n*/*n*	*n*		*n*				
Chen^52^	2020	2016–2019	54	54.7±13.9	28/26	39	Whipple	15	Total^†^	Retrospective controlled	2c	8
Qin^53^	2020	2007–2018	28	10.8–12	ND	6	PPPD	22	Total	Retrospective controlled	2c	8
Jiang Yu^54^	2018	2016–2016	68	47±14.7	21/47	34	PPPD	34	Total^†^	Prospective controlled	2b	9
Li Yatong^55^	2017	2008–2014	62	49.5/49.8	29/33	42	PPPD	20	Total	Prospective controlled	2b	8
Perinel^56^***	2014	2007–2012	39	59/64	17/22	24	PPPD	15	Total 13 Partial 2	Retrospective controlled	3a	9
Liu^57^	2013	ND	57	49.3/46.2	34/23	31	PPPD	26	Partial	Prospective controlled	2b	9
Gong^58^	2013	1998–2011	58	40±12 45±10	22/36	40	PPPD	18	Partial	Retrospective controlled	2c	8
Pedrazzoli^59^	2011	1991–2008	64	54 61	33/31	37	PPPD	27	Total 23 Partial 4	Prospective controlled	2a	9
Fujii^60^	2011	1991–2009	132	51/62.4	80/52	55	PPPD	77	Total	Prospective controlled	2a	9
Busquets^61^**	2010	1989–2006	62	51/46	42/20	41	Whipple17 PPPD 24	21	Partial	Prospective controlled	2b	9
Horiguchi^62^	2010	ND	40	67/59	22/18	19	PPPD	21	Total	Prospective controlled	2b	9
Lee^63^	2010	1995–2007	100	56	42/58	70	PPPD	30	Total	Prospective controlled	2a	9
Aspelund^64^*	2005	1997–2001	31	56/44	17/14	7	Whipple	24	Partial	Retrospective controlled	2c	8

*12 patients with additional PMD drainage

**3 patients with TM enucleation included

***In 9 and 5 patients, who underwent DPPHR or PPPD respectively, total pancreatectomy was done

^†^Laparoscopic or robotic-assisted DPPHR

### Duodenum-Preserving, Total or Partial Pancreatic Head Resection

Partial pancreatic head resection DPPHRp (type I) was performed when tumor size and the proposed biological nature of the neoplasm necessitated tissue resection extending beyond the pancreatic main duct. DPPHRp does not require resection of the duodenum and/or common bile duct (CBD); the tissue outside of the tumor wall of the ventral or dorsal pancreatic head is preserved (Fig. S2A).

DPPHRt involves resection of the pancreatic head with the tumor, while conserving the pancreatic neck, intrapancreatic CBD, and duodenum (Fig. S2B, type II). A subgroup of DPPHRt comprises patients who underwent resection of the peripapillary segment of the duodenum (DPPHRt + sd) and resection of the intrapancreatic CBD (Fig. S2C, type III). A few patients are included in the DPPHRt group, who underwent near total pancreatic head resection by conserving, after resection of the uncinate process, some suprapapillary pancreatic tissue of the groove of the pancreas. Reconstruction technique was predominantly pancreaticojejunostomy (DPPHR type I–III) or, less frequently, pancreatico-gastrostomy (DPPHR type III) or duodeno-duodenostomy and pancreatico-duodenostomy (DPPHR type III).

### Data Extraction Process

The presented data are based on a selective evaluation of 34 studies dealing with DPPHR published between 1994 and 2020. Data extraction from each study was conducted independently by two authors (HB, BP) according to the lists of pre-specified selection criteria. To evaluate the intraoperative and early postoperative outcomes, the following criteria were used for analysis: operating time, intraoperative blood loss, intra- and postoperative blood transfusion, postoperative overall and serious or severe surgery-related morbidity leading to invasive re-interventions, re-operation, in-hospital mortality, re-hospitalization, tumor size, frequency and grade of pancreatic and biliary fistula, and length of postoperative hospital stay. Pancreatic fistulae were classified according to the guidelines of the International Study Group for Pancreatic Fistula.^65^ Biliary fistulae were recorded according to the recently published definitions.^66^ DGE was defined following the international guidelines for DGE.^67^ Reports published before the presentation of the guidelines do not fully reflect the classifications. The surgical techniques, partial and total pancreatic head resection, and type of reconstruction were listed separately for each report. Severe early postoperative complications were defined using the Clavien-Dindo -classification III a+b. Surgical complications IIIa were listed as serious and IIIb as severe complications necessitating re-intervention or re-surgery.^68^ Complications requiring endovascular-radiologic, endoscopic, laparoscopic, or transhepatic reintervention for post-pancreatectomy bleeding (PPH), gastrointestinal bleeding (GIH), intraabdominal abscess, pancreatic fistula, large fluid collection, and biliary fistula leading to jaundice or cholangiosepsis were separately listed as surgery-related serious complications. All publications presented data on frequency of pancreatic and biliary fistula. However, only studies published after 2005 used the classification of POPF A, B, and C according to the International Study Group of Pancreatic Fistula definition. The presence of each criterion was given in relation to the total number of patients reported. The variations in the denominators of patients in the tables reflect some reports lacking data of the specific criteria and were therefore not included in the respective statistical calculations, except for meta-analysis. The final histology of the tumors was listed separately including IPMN, MCN, SPN, SCA, and pancreatic non-functional and functional PNETs as well as periampullary tumors. Chronic pancreatitis and other cysts and tumors were included in most reports and additionally listed. Advanced pancreatic cancer, preoperatively considered benign tumor, but identified by frozen section investigation and /or by final histological diagnoses, was listed under “other” tumors, as presented in the respective publications.

Most patients with advanced cancer, identified by frozen section, experienced either intraoperative conversion to PD or early postoperative oncological re-surgery by PD and/or chemotherapy during the index hospitalization.

The indication for DPPHR or PD was based on the presence of abdominal symptoms in 87% of the patients included in the review and meta-analysis. All tumors were considered preoperatively to be of benign nature, except some patients with papillary/ampullary tumors. The final oncological diagnosis of the tumors was based on the histopathology given in the reports. Periampullary tumors were subdivided into tumors of the papilla and ampulla, the peripapillary duodenum, and the peripapillary CBD, including pancreatico-biliary maljunctions. Evaluating long-term outcomes, the data of time and reason for late mortality during the reported follow-up period were separately listed including data of 543 of 654 DPPHR patients (83.0%). Seven authors were contacted by e-mail to clarify the cause and type of postoperative interventions and histopathological classification of the tumors, which were lacking in their respective publication.^38, 40, 50, 51, 53, 55, 58^ The reported time period covers 27 years. The criteria POPF B+C, DGE, rehospitalization, and the definition of main duct (MD) and branch duct (BD) IPMN are incompletely reported because the clinical, histopathological, and radiological criteria were established as guideline metrics only in recent years.

### Statistical Analysis

All analyses were conducted using R for statistical computing (version 4.0.2, www.r-project.org, package meta). Continuous variables were expressed as mean and standard deviation (SD), and categorical variables were presented as absolute frequencies and percentages. Explorative statistical testing of the DPPHR subgroups (total vs. partial resection) was performed using the chi-square test. Statistical significance was set at *p* < 0.05. For the meta-analytic approach, the odds ratio (OR, Mantel-Haenszel method) was used for all considered dichotomous outcomes.^69^ All effect estimates were presented together with their 95% confidence intervals (CIs). To assess the extent of between-study heterogeneity, the *I*^2^ statistic was evaluated, leading to the application of a fixed-effects model where *I*^2^ was <40%; otherwise, a random-effects model was used. A graphical representation of the results was based on forest plots. To determine whether significant publication bias had to be assumed, funnel plots were additionally created.

## Results

### Study Groups

The analysis was based on 34 good- to high-quality cohort studies presenting data of 654 patients following DPPHR (Tables 1 and 2). A total of 445 patients included in the meta-analysis underwent PD for benign tumors, premalignant neoplasms, or low-risk malignant periampullary tumors. The systematic review was performed by analyzing the DPPHR-related data of all patients of the 34 cohort studies. DPPHRt was reported for 462 patients and DPPHRp for 192 patients. The meta-analysis was based on data from 13 controlled studies, including the control group of patients who underwent PD. The results of 350 patients following DPPHR (255 patients underwent DPPHRt, 95 patients DPPHRp) were compared with 445 patients following PD (87 patients underwent Whipple resection, 358 patients PPPD) in the meta-analysis.

### Assessment of Methodological Quality of Studies

The systematic review and meta-analysis were based on 21 cohort studies in the review group (Table 1) and 13 studies in the meta-analysis group (Table 2). In total, 19 studies were controlled cohort studies, of which 13 were prospective and 6 retrospective reports. Fifteen reports were without a control group, nine of them prospective studies. The critical appraisal for methodology revealed 24 studies with evidence level 2 and ten studies with evidence level 3. Evidence level 2 certifies a good quality cohort study. Additionally, the NOS score was used to assess the quality of all cohort studies which enabled an objective evaluation of the most basic quality aspects of non-randomized studies. Twenty-seven cohort studies elicited a score of ≥ 8; the mean NOS score was 8.1, which indicated a good quality of the cohort studies.

### Results of Baseline Data

The baseline data of the 34 cohort studies comprising 1099 patients are presented in Tables 1 and 2. The 34 studies comprised data of 654 patients following DPPHR and 445 patients following PD. Twenty-three studies were published between 2010 and 2021. In the review group (Table 1), the mean age of the patients was 50.1 years (SD ± 13.0) and in the group of the meta-analysis, the mean age was 51.2 years (SD ± 11.3) (Table 2). The gender relationship M/F was 1.5 across all studies. In two studies, results were reported after the use of DPPHR in adolescents and children, predominantly for SPN.^33, 52^

### Results of Tailored use of Duodenum-Preserving Pancreatic Head Resection and Reconstruction of the Gastrointestinal Tract

In total, 462 patients (70.6%) underwent DPPHRt and 192 (29.4%) DPPHRp (Table 3). Tumor size of the DPPHRt group of 3.7 cm was significantly larger (*p*<0.001) than that in the DPPHRp group (2.9 cm). A complete preservation of the duodenum was experienced by 290 patients of the DPPHRt group. In total, 172 patients underwent resection of the peripapillary duodenum and the CBD (DPPHRt + sd). In 192 patients, who underwent DPPHRp, the duodenum and intrapancreatic CBD was preserved, except in 7 patients, who experienced additional CBD resection. DGE, length of postoperative hospital stay, and in-hospital mortality were significantly lower following DPPHRp compared to DPPHRt (Table 3). Gastrointestinal (GI)-tract reconstruction was performed with an end-to-end anastomosis of the duodenum in 172 patients; 199 patients had an anastomosis of the CBD with the duodenum. GI-tract reconstruction of the left pancreas was performed in 381 patients with an excluded jejunal loop, with the stomach in 198 patients, with the duodenum in 50 patients and as a duct-to-duct anastomosis of the pancreatic main duct in 15 patients. In 15 patients, a duodenum-preserving total pancreatectomy was performed, of them in 10 patients with conservation of the spleen. (Table 3).

**Table 3:** Early postoperative morbidity of 654 patients following DPPHR for benign tumors of the pancreatic head

Type of DPPHR*	Patients	TM size	Early postoperative morbidity						
			Pancreatic fistula** POPF *n*/*N*	DGE*** total *n*/*N*	Biliary fistula *n*/*N*	Reoperation *n*/*N*	Length of postop. hospital stay (LHS)	In-hospital mortality *n*/*N*	Re-hospitalization *n*/*N*
	*N*	cm	%	%	%	%	Days/mean	%	%
Total head resection	462	3.7±0.62^†^	110/462 23.8%	43/359^†††^ 11.9%	20/411^†††^ 4.8%	11/424^†††^ 2.6%	27.9^††^	3/462 0.65%	5/184^†††^ 2.7%
Partial head resection	192	2.97±1.01^†^	33/164^†††^ 20.12%	7/112^†††^ 6.2%	6/167^†††^ 3.5%	4/180^†††^ 2.2%	16.7^††^	0/192 0%	2/117^†††^ 1.7%

*Laparoscopic and robotic-assisted DPPHR: 81 patients

**POPF since 2006: B/C

***DGE since 2007 B+C:

Before: gastric tube removal after 7th to 10th postop. day

^†^Tumor size: *p* < 0.001

^††^LHS: DPPHRp vs. DPPHRt *p*=0.02

^†††^No data: POPF: ^36, 38^

DGE: ^24, 32, 36, 37, 42–44, 48, 50, 51, 58, 59^

Reoperation: ^43, 48, 53^

Rehospitalisation: ^24, 31, 32, 36, 38, 45–48, 50, 51, 56, 59–64^

**Anastomosis with left pancreas (p):

p-jejunum (e-s/s-s): 381 patients

p-stomach (e-s): 198 patients

p-duodenum (e-s): 50 patients

p-duct-to-duct (e-e): 15 patients

DPTP: total pancreatectomy 10 patients

Of the 445 patients, who underwent PD for benign tumors of the pancreatic head, Whipple resection was performed in three studies (87 patients) and PPPD in ten studies (358 patients) (Table 2). Of those undergoing PD, pancreatico-jejunostomosis was performed in 280 patients and pancreatico-gastrostomosis in 128 patients; two studies reported PPPD but not the type of pancreatic anastomosis.^53, 57^ The final histopathologic diagnosis revealed 420 patients with cystic neoplasm and 83 patients with PNET. Thirty-four patients displayed tumors of the papilla/ampulla or peripapillary duodenum or peripapillary CBD and/or maljunction of the pancreatic and biliary ducts. “Other” tumors were reported for 111 patients (Table 4). Under “other” tumors, which were operated with the diagnosis of a benign neoplasm, 15 patients presented with the histopathology of advanced adenocarcinoma intraoperatively by frozen section and/or by the final histopathology. These patients were listed in the section “others”; nine of them experienced conversion to classical PD or resurgery PD during the index hospitalization or DPPHRt plus adjuvant chemotherapy.

**Table 4:** Final histopathology of 654 patients following DPPHR for benign tumors, cystic, and neuroendocrine neoplasms and periampullary tumors of the pancreatic head

	Patients	Cystic neoplasms				PNETs	Periamp. tumors/ papilla/ampulla duodenum/CBD^†††^	Other tumors	
	*N*	IPMN^††^ *n*	MCN *n*	SPN** n	SCA *n*	Non-funct./functional *n*	*n*	Chronic pancreatitis *n*	“Others”/malignant TM^††††^ *n*
Total DPPHR	462	195*	39	48**	36	46***	32	47	19
Partial DPPHR^†^	192	38*	27	12**	25	37***	2	39	6

*IPMN: DPPHR total vs. partial *p*<0.0001

**SPN: DPPHR total vs. partial *p*<0.0351

***functional PNETs predominantly sporadic insulinomas

^†^Missing data from 6 patients ^48^

^††^IPMN: predominantly branch-duct type, when histologically differentiated

^†††^Papillary/ampullary TM 16 pats. (10 benign TM, 6 carcinoma in adenoma), Duodenal TM 5 pats. (1 benign TM, 4 duodenal carcinoid), Peripapillary CBD TM 6 pats. (4 benign TM, 2 carcinoma), Duct maljunction 7 pats. (5 benign, 2 biliopancreatic maljunction + *T*_1_ CBD cancer)

^††††^Includes 9 pats. with IPMC and 2 pats. with PDAC, 3 pats. with CBD cancer, 1 pat. with MCN carcinoma, 2 pats. with metastasis renal cell cancer, 1 pat. with metastasis of ileal carcinoid

### Results of Early Postoperative Morbidity Following Total or Partial DPPHR

The overall morbidity rates following DPPHRt and DPPHRp were 40.7% and 39.5% respectively. The frequencies of pancreatic fistula, biliary fistula, DGE, and reoperation were on the same level comparing total and partial pancreatic head resection (Table 3). In 115 patients (17.6%), a laparoscopic or robotic-assisted DPPHR was performed.^31, 32, 34, 37, 38, 52, 54^ Following DPPHRt, in-hospital mortality was 0.65% and 0% following DPPHRp (Table 3).

DPPHRt was more frequently used for surgical treatment of IPMN and SPN. For functional PNETs, DPPHRt was more frequently applied for patients with sporadic insulinoma (Table 4). Non-functional PNETs were predominantly treated with DPPHRp. Of the 233 patients with the final diagnosis of IPMN, predominantly BD-IPMN was histologically verified, when the guidelines for IPMN subgrouping were applied. Almost all patients with periampullary tumor underwent a total DPPHR (DPPHRt + sd); in all patients, resection of the peripapillary segment of the duodenum and CBD was performed. Final histopathology revealed an inflammatory tumor of the pancreatic head in chronic pancreatitis in 13.1% of 654 patients, preoperatively considered a benign solid tumor. The section of “other tumors” (Table 4, “others”) included 15 of 25 patients who in the final histopathology displayed advanced cancer (pancreatic ductal carcinoma in two patients, IPM cancer in nine patients, CBD cancer in three patients, and MCN cancer in one patient). Additionally, two patients underwent DPPHRt for renal cell cancer metastasis and one patient for ileal carcinoid metastasis. In four patients, a conversion to PD was performed, in three patients subsequently a PD during the index hospitalization and in two patients an adjuvant chemotherapy. The mean follow-up time after DPPHRt was 46.9 months and 53.5 months after DPPHRp

### Results of Meta-analysis Comparing DPPHR and PD for Early Surgery-Related Postoperative Morbidity

**Fig. 2:**
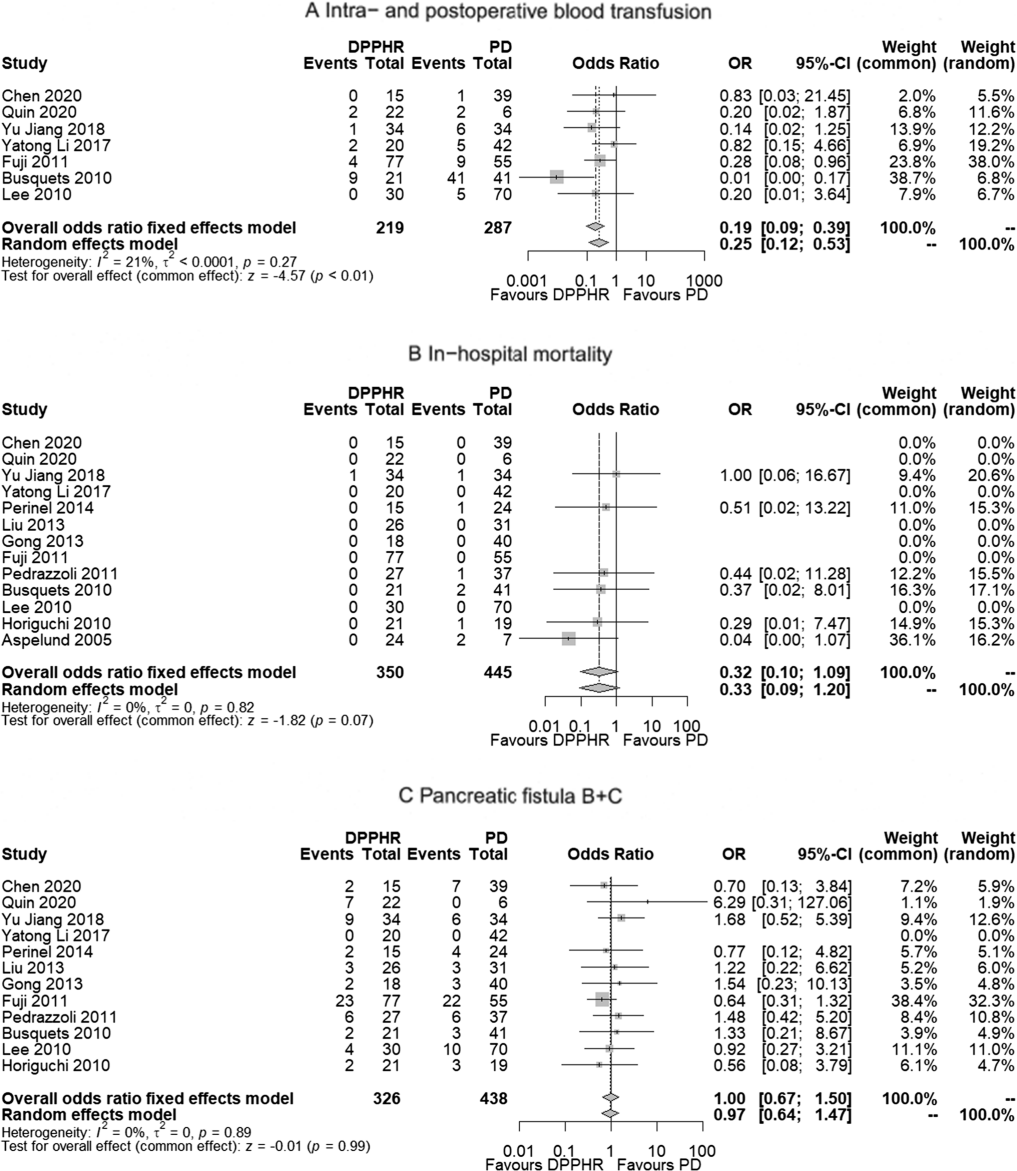

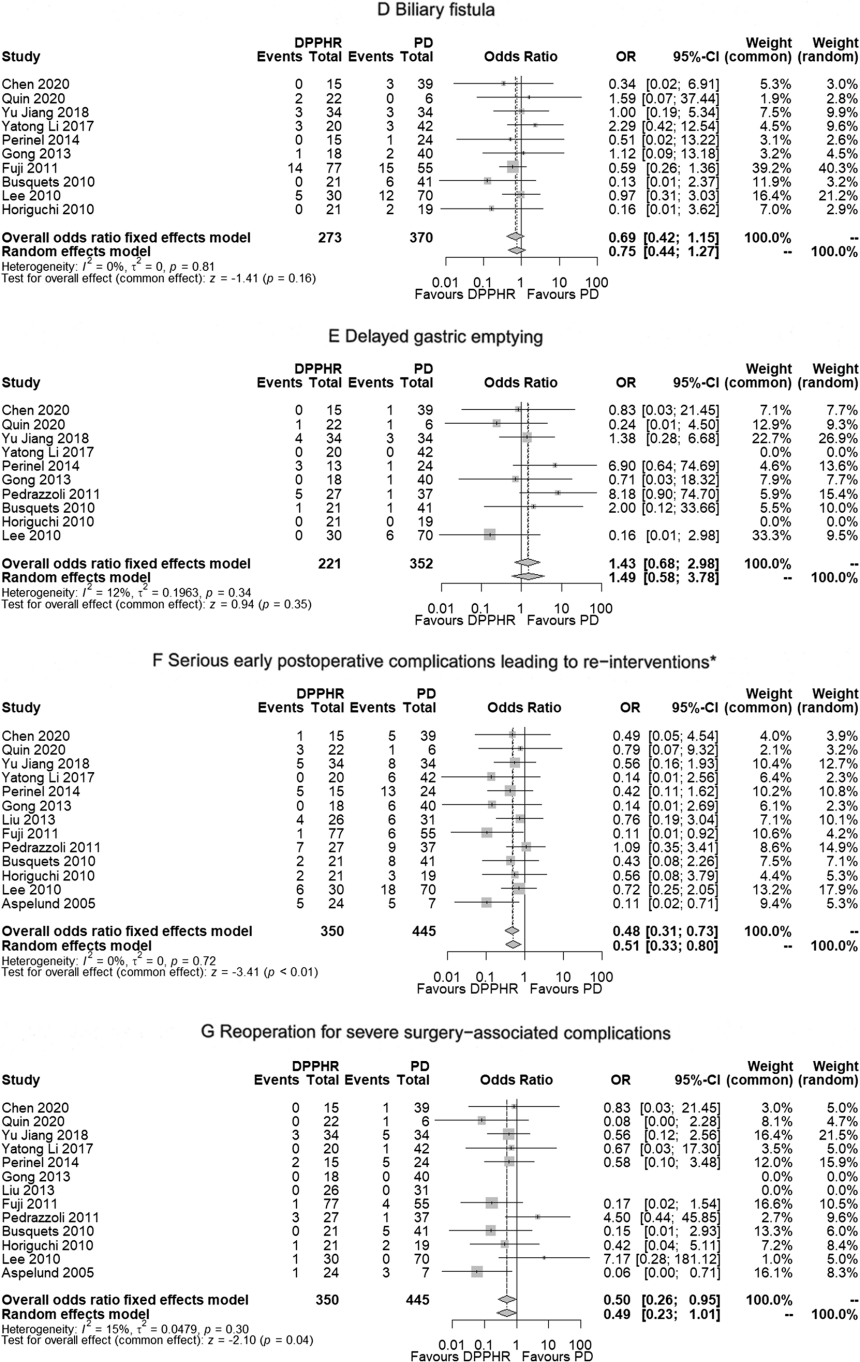
**A**–**G** Forest plots of postoperative surgery-related complications following DPPHRt compared to PD. **A** Intra- and postoperative blood transfusion. **B** In-hospital mortality. **C** Pancreatic fistula B+C. **D** Biliary fistula. **E** Delayed gastric emptying. **F** Serious early postoperative complications leading to reinterventions* *(interventional treatment for PPH, GIH, large fluid collection, intra-abdominal abscess, re-drainage, break of anastomosis, biliary fistula + cholangitis/sepsis). **G** Reoperation for severe surgery-associated complications

The meta-analysis was based on 13 studies published between 2005 and 2020 comparing postoperative data following DPPHRt or PD. The procedure-related, intraoperative metrics were less frequently observed after DPPHRt compared to PD: mean operation time 332 min vs. 369 min (*p*=0.35); mean estimated blood loss 368 ml vs. 432 ml (*p*=0.44). The need for intra- and postoperative blood transfusion was 19/222 patients vs. 69/287 patients, being significantly lower in the DPPHR group (OR 0.20, 95%CI 0.10–0.41, *p*<0.01) (Fig. 2A). The in-hospital mortality rate following DPPHRt compared to PD was 1 of 350 patients (0.28%) and 8 of 445 patients (1.79%) respectively (*p*=0.07) (Fig. 2B). Following DPPHRt, pancreatic fistula B + C was observed in 62 of 326 patients (19.0%) and following PD in 67 of 438 patients (15.29%), (*p*=0.99) (Fig. 2C). DGE following DPPHRt and PD was observed in 28 of 273 patients (10.25%) and in 47 of 370 patients) (12.70%), respectively (*p*=0.16) (Fig. 2E). Biliary fistula was observed following DPPHRt and PD in 14 of 221 patients and in 15 of 352 patients, respectively (*p*=0.35) (Fig. 2D).

Comparing baseline data after DPPHR and PD analyzed in the meta-analysis, age (mean 48.8 vs. 52.5 years), gender M/F (1.2/0.8 vs. 1.3/0.95), frequency of cystic neoplasm (218 vs. 243 patients), PNET (48 vs. 59 patients), periampullary neoplasm (17 vs. 37 patients), and chronic pancreatitis (13.7% vs. 13.9%) were not significantly different in both groups. Tumor size was slightly larger in the DPPHR group (mean 3.8 vs. 3.4 cm). In the final histopathology, advanced cancer was more frequently found in the PD group (8.1% vs. 4.0%) (p<0.029) due to advanced peripapillary cancer. Approximately two thirds of IPMN included in the DPPHR group revealed BD type of IPMN, whereas in the PD group MD and mixed type prevailed.

Interventions for serious surgery-related complications following DPPHRt, comprising interventional treatment for PPH, GIH, intraabdominal abscess, large peripancreatic fluid collection, and biliary fistula/cholangitis leading to immediate interventional safety measures, were significantly less frequently observed following DPPHR in 41 of 350 patients (11.7%) than following PD in 93 of 445 patients (20.9%) (OR 0.48, 95% CI 0.31–0.73, *p*<0.01) (Fig. 2F). Reoperation was less frequent following DPPHR (12 of 350 patients; 3.4%) compared to 29 of 445 patients following PD (6.5%) (OR 0.50, 95% CI 0.26–0.95, *p*=0.04) (Fig. 2G).

**Fig. 3:**
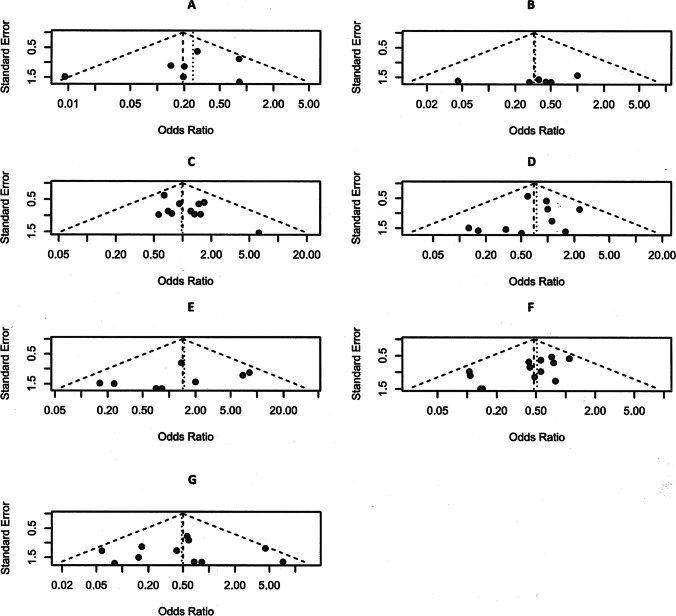
(**A**–**G**) Funnel plots. (**A**) Comparison of intra- and postoperative blood loss following DPPHRt versus PD. (**B**) In-hospital mortality. (**C**) Frequency of pancreatic fistula B+C. (**D**) Frequency of biliary fistula. (**E**) Delayed gastric emptying. (**F**) Frequency of reintervention for serious, local complications. (**G**) Frequency of reoperation for severe surgery-associated complications

Reinterventions for serious, local complications necessitating radiologic, angiographic, endoscopic, transgastric, transabdominal, ERC, or transhepatic bile duct interventions were significantly more frequently observed following PD. Comparing 350 DPPHR and 445 PD patients, the frequency of adverse events was for PPH 13 vs. 28 (*p*=0.07), GIH 6 vs. 15 (*p*=0.06), intraabdominal abscess 9 vs. 32 (*p*=0.02), large fluid collections 1 vs. 9 (*p*=0.63), and biliary fistula/cholangiosepsis 9 vs. 10 interventions. In total, 38 of 350 DPPHRt patients vs. 94 of 445 PD patients experienced reintervention following serious complications (*p*=0.01). All results were created on the basis of a fixed effects model due to the absence of study heterogeneity (*I*^2^ = 0% in Fig. 2A, C, D) and a low level of heterogeneity in Fig. 2B and E. There was no reference for publication bias as demonstrated by funnel plots (Fig. 3(A–G)).

## Discussion

Parenchyma-sparing pancreatic head resection has evident advantages for patients undergoing surgery for benign tumors and premalignant cystic and neuro-endocrine neoplasms. The meta-analysis comparing the results of DPPHR and PD displayed a very low in-hospital mortality of one of 350 patients (0.28%) following DPPHR (*p*=0.07). Moreover, no hospital mortality was observed in the 192 patients undergoing DPPHRp. The frequencies of intra- and postoperative blood transfusion, reintervention for serious early postoperative complications, and reoperation were significantly less after DPPHR. Operating time, intraoperative and postoperative blood loss, and DGE were lower after DPPHRt, but not statistically significant different compared to PD.

PD for benign tumor or cystic neoplasm is associated with a considerable risk of surgery-related complications and mortality due to this being a multi-organ resection procedure. In this group of the 445 patients of the meta-analysis, the in-hospital mortality following PD was 1.79%. A few high-volume centers for surgical treatment of benign tumors of the pancreas reported an in-hospital mortality after PD below 2.0%^2, 70^; however, recently published results of large investigations revealed an in-hospital mortality after PD for benign tumors still of 2–6%.^3, 71^ The risks for PD-associated early postoperative complications, late-outcome metabolic morbidity^22, 72^ and new onset of DM,^11, 13^ conversion of preoperative non-insulin dependent diabetes to insulin-dependent treatment of up 40%,^73^ and high degree of pancreatic exocrine insufficiency of up to each second patient^74^ pose a substantial challenge to surgeons with respect to decision-making and preoperative counseling of patients suffering a benign pancreatic tumor.

Duodenum-preserving total pancreatic head resection with complete preservation of the duodenum and the intrapancreatic common bile duct is technically demanding, necessitating a meticulous dissection of the duodenum and common bile duct from the pancreatic head tissue with the focus on maintenance of the blood supply to the peripapillary duodenum via superior-posterior and inferior-anterior gastroduodenal branches of the pancreatico-duodenal arterial arcade.

Comparing the surgical techniques of DPPHRt and PD revealed clear similarities in the operative steps, which may explain the comparable pattern of pancreatic and biliary fistula following both procedures. On expositions of the pancreatic head and processus uncinatus, the portal and superior mesenteric veins were identical in both procedures (Fig. S2). Dissection of the pancreatic head along the duodenal wall by transection of the mesoduodenum to separate the duodenum from the processus uncinatus towards the papilla of Vater and transection of the suprapapillary pancreatic groove tissue from the duodenal wall are specific surgical steps of DPPHRt. Conservation of the anterior branch of the inferior pancreatico-duodenal artery, which frequently runs close to the uncinate process along the ventral wall of the duodenum, and conservation of the posterior branch of the superior pancreatico-duodenal artery are difficult surgical steps of the DPPHRt procedure. Dissection of the intrapancreatic CBD segment, which varies in length, implies the risk of injuring the CBD wall. The frequency of biliary fistula of 4.5% following partial and total DPPHR (Table 3) is caused by duct wall injury or ischemic lesion of the prepapillary, intrapancreatic CBD segment. To avoid the development of biliary fistula, CBD stricture, or cholangitis, it is important to preserve the posterior branch of the superior pancreatico-duodenal artery during dissection of the intrapancreatic segment of the CBD. An ischemic trauma of the peripapillary duodenal wall due to dissection of the pancreaticoduodenal arteries was observed only in three of the 462 patients, who underwent DPPHRt.^45, 61^ To avoid biliary leakage, the application of indocyanine green (ICG) fluorescence imaging is recommended to delineate the bile duct intraoperatively. ICG enables real-time identification of biliary anatomy intraoperatively to avoid duct injury.^75, 76^

The frequency of biliary fistula following DPPHR was notably higher, but statistically not significantly different than following PD (6.33% vs. 4.26%) (Fig. 2D). Complete dismantling dissection of the intra pancreatic segment of the CBD from pancreatic tissue as performed in 221 DPPHRt patients explains the increased risk for injuring the CPD and the frequency of biliary fistula (Fig. 2E). Incomplete resection of pancreatic head tissue of the supra-papillary groove pancreas, while performing a nearly total DPPHR, increases the risk for POPF, which explains the high fistula rate in some institutional series included in this analysis.^53, 54, 59^ For subtotal pancreatic head resection conserving a shell-like rest of the pancreatic tissue close to the duodenal wall, a second pancreatico-jejunostomosis (side to side) clearly reduces the fistula rate, as shown by DPPHR for chronic pancreatitis.^77^

Duodenum-preserving pancreatic head resection for benign tumors, cystic neoplasms, and neuroendocrine neoplasms should be performed in high-volume centers for pancreatic surgery.

### Tailoring DPPHR

The decision to use local tumor extirpation by DPPHRp, similar to the Berne modification^78^ or DPPHRt, is determined by tumor size, but more importantly by the biological nature of the neoplasm, pathology of the pancreatic main ducts in the head, and tumor abutment to the intrapancreatic segment of the CBD and the duodenal wall. The advantages of DPPHRp compared to DPPHRt are lower frequencies of biliary fistula, DGE, and in-hospital mortality and a shorter hospital stay, as shown in Table 3.

Tumor size in the group of DPPHRt was significantly larger than it was in the DPPHRp group. Large size of tumor and the proposed preoperative diagnosis confirmed by intraoperative application of IUS and frozen section investigation explain the most frequent decision to apply DPPHRt for IPMN, MCN, and SPN, and for periampullary tumors. Because the pathohistological guidelines differentiated between BD- and MD-IPMN, DPPHRt was preferentially applied for BD-IPMN, whereas for MD-IPMN a PD was performed. For non-functional PNETs of the pancreatic head, a local, limited tumor resection (DPPHRp) was preferentially applied. PNETs larger than 3 cm in maximum size or the presence of sporadic insulinoma were the most frequent indications for DPPHRt.

Tumor enucleation is considered the first-choice surgical treatment for small tumors of 2-cm size of the pancreatic head.^79, 80^ However, in the pancreatic head, involvement of the pancreatic main ducts and the risk of duct injury limit the use of tumor enucleation of the pancreatic head. The risk of high-volume pancreatic fistula is considerably increased when the main duct in the pancreatic head is injured.^81^ Pancreatic main duct opening during enucleation has the risk for high volume pancreatic fistula and leads to a complicated clinical course and to extended hospitalization. The high frequencies of POPF B + C fistula after TE limit its use for cystic and neuro-endocrine neoplasms of the pancreatic head.^82^ For patients with neuro-endocrine tumors, predominantly DPPHRt was performed. Sporadic insulinoma were the prevalent diagnosis among functional PNETs (Table 4). DPPHR enables a systematic lymph node sampling around the pancreatic head additionally to the extirpation of a neuro-endocrine neoplasm for the staging of PNET.^55^

Periampullary tumors are rare and frequently transferred to surgical treatment following multiple endoscopic interventions. A total of 32 patients with periampullary tumor pathologies was treated with DPPHRt, including segment resection of the peripapillary duodenum and CBD resection. As documented in Table 4, the advantage of DPPHRt with resection of the peripapillary duodenum and the pancreatic head for patients with villous adenoma and T_1_ cancer of the papilla is that DPPHRt + sd offers a cure for patients without a high risk for surgery-related morbidity, incomplete resection, or fear of tumor recurrence. Most neoplastic tumors were low-risk adenoma of the papilla and ampulla; non-neoplastic indication for DPPHRt was mostly bilio-pancreatic duct maljunction and pancreas divisum causing periductal an inflammatory tumor. In total, for benign tumors, the concordance of the preoperative and final histopathology of the resected tumors was 88.7%.^83^

This systematic review and meta-analysis has clear limitations. Generally, the inclusion of cohort studies based on a small number of patients and on patients with inflammatory tumor increases the risk of bias and limits the conclusion. Four of 13 studies used for meta-analysis were retrospective, controlled investigations. The comparison of results after DPPHRt or PD were published with one exception in the past 11 years. However, the data derived from the studies in the review group comprise a reporting period of 27 years. The inclusion of non-comparative studies does not considerably add to the body of evidence. During the last 10 years, the management of early postoperative complications following pancreatic head resection regarding non-surgical treatment of serious complications has involved the use of intravascular, radiologic, endoscopic, transhepatic, and laparoscopic techniques which have developed to routine interventions, avoiding severe complications with the need for reoperation. This may have influenced the evidence of the overall results of the presented review and meta-analysis. The results of randomized, controlled trials are warranted to establish with high-quality clinical evidence the advantages and/or drawbacks of DPPHRt compared to PD. A randomized, controlled comparison of DPPHRp and tumor enucleation for benign neoplasms of the pancreatic head, including small neuroendocrine tumors, is greatly needed. With respect to the long-term oncological outcome of patients with premalignant cystic and neuroendocrine neoplasms, the data are separately analyzed and under publication.^83^

## Conclusion

Local, parenchyma-sparing resection of the pancreatic head for benign and premalignant tumors leads to cure of patients while preserving the duodenum, gastric antrum, and biliary and pancreatic tissues. Assessment of severe and serious complications following DPPHR and PD revealed a significant lower risk for reoperation and reintervention following DPPHR caused by break of pancreatic anastomosis or serious post-pancreatectomy and gastrointestinal bleeding, intraabdominal abscess, large fluid collection, jaundice, and cholangitis-associated biliary fistula. The low in-hospital mortality rate of 0.49% after DPPHR reflects the limited tissue trauma. Tailored use of total and partial DPPHR contributes to the low level of surgery-associated early postoperative complications. DPPHRt is a technically demanding procedure with respect to the maintenance of blood supply of the peripapillary duodenum. Undergoing surgery for benign tumors and premalignant cystic and neuro-endocrine neoplasms of the pancreatic head, DPPHR has the potential to become the first-choice treatment.

### Supplementary Information

Below is the link to the electronic supplementary material.

Supplementary file1 (PDF 183 KB)

## Declarations

The first author is originator of the procedure duodenum-preserving pancreatic head resection.
