# Supplementary material for: Duodenum-Preserving Pancreatic Head Resection for Benign and Premalignant Tumors—a Systematic Review and Meta-analysis of Surgery-Associated Morbidity
Source: J Gastrointest Surg. 2023 Sep 5;27(11):2611–27. doi: 10.1007/s11605-023-05789-4 (PMC10661729; doi:10.1007/s11605-023-05789-4)

Parenchyma-sparing pancreatic head resection - Beger et al.

Supplemental Material **Figures S2A - S2C**

Fig. S2A – Partial DPPHR (DPPHRp, Type I) (192 patients)
Partial pancreatic head resection; reconstruction with side-to-side pancreaticojejunostomosis

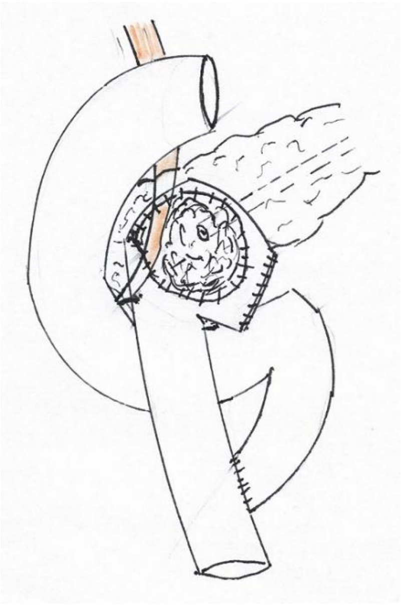

Fig. S2B – Total DPPHR (DPPHRt, Type II) (290 patients) with preservation of the duodenum and the intrapancreatic common bile duct; reconstruction using the first jejunal loop

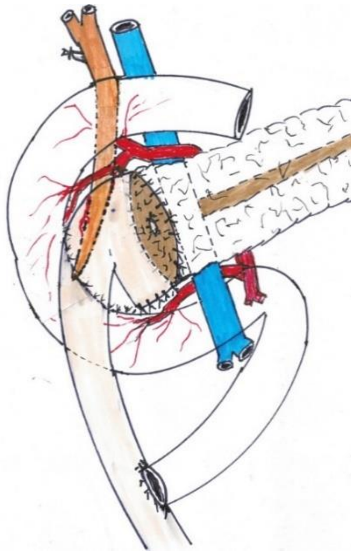

Fig. S2C – Total DPPHRt (DPPHRt + sd, Type III) (172 patients) Total DPPHR with segment resection (sd) of the peripapillary duodenum and resection of the intrapancreatic common bile duct. Reconstruction with biliary anastomosis (e-s) duodenum anastomosis (e-e) and
Pancreatico-jejunum anastomosis (e-s).

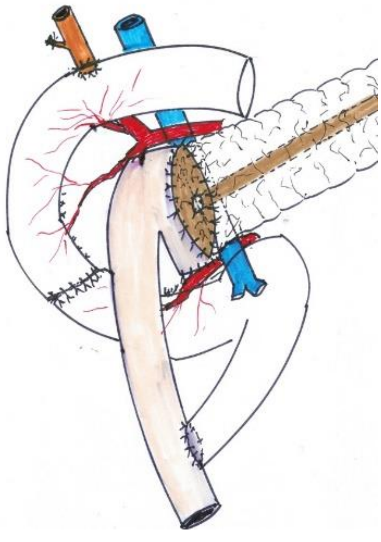

Supplement: Supplementary file 1 — Supplementary file1 (PDF 183 KB) [file 11605_2023_5789_MOESM1_ESM.pdf]
